# Amlodipine/Valsartan (Avsar®): Efficacy in Hypertensive Patients - A Real World Observational Study (ALERT)

**DOI:** 10.7759/cureus.8174

**Published:** 2020-05-17

**Authors:** Khalid M Khan, Somia Iqtadar, Mahmood Nasir, Anum S Siddiqui, Atiq Rehman

**Affiliations:** 1 Internal Medicine: Gastroenterology, Jinnah Hospital, Lahore, PAK; 2 Internal Medicine, King Edward Medical University & Mayo Hospital, Lahore, PAK; 3 Internal Medicine: Gastroenterology, Gulab Devi Hospital, Lahore, PAK; 4 Miscellaneous, Pharmevo Pvt. Limited, Karachi, PAK; 5 Cardiology, Clinision, Karachi, PAK

**Keywords:** hypertension, amlodipine, valsartan, blood pressure

## Abstract

Objectives: Hypertension is a significant public health problem and one of the major noncommunicable diseases at the endemic level in Pakistan. This study was done to determine the efficacy of amlodipine/valsartan (Aml/Val) once-daily dose in reducing blood pressure (BP) after eight weeks of therapy.

Methods: This study is an open-labeled observational study carried out for a period of 12 months. Some 769 participants of either gender between the ages of 18 and 70 years selected after taking written informed consent had a BP of >139/89 mmHg (not controlled) on monotherapy with a minimum 30 days of treatment. Therapy to control their high BP was initiated with Aml/Val (Avsar^®^, PharmEvo Pvt Ltd, Karachi, Pakistan) at the time of their enrolment in the study. Pregnant females and patients with secondary hypertension were excluded. Data were analyzed using SPSS version 20.0 and chi-square test was used for inferential analysis. p-values less than 0.05 were considered significant.

Results: At the end of week one, less than half of the patients achieved the desired level of BP while the majority achieved this level by the end of the study. Some 75.6% patients achieved targeted BP with Aml/Val 80/5 mg tablet, 18.5% achieved targeted BP with Aml/Val 160/5 mg tablet, and 5.9% achieved the targeted BP with Aml/Val 160/10 mg tablet at the end of the eighth week. The compliance rate was 99.2% at the first week, 98.9% at the fourth week, and 99.9% at the eighth week of treatment.

Conclusion: Our study concluded that Aml/Val (Avsar) combination therapy was very effective in controlling BP among patients who were uncontrolled with other monotherapies for at least one month.

## Introduction

Hypertension is a significant public health problem, with a worldwide prevalence of 40.8% and a controlled rate of 32.3%. It is a major risk factor for several serious health conditions, including cardiovascular disease (CVD), cerebrovascular disease, and chronic kidney disease. Worldwide, 9.4 million deaths are attributed to complications from hypertension, including 45% of all deaths due to coronary artery disease and 51% of all deaths due to stroke [1]. In around 80% of deaths due to cardiovascular causes in low-income countries, hypertension is highly common [2]. Two major studies, one based on a National Health Survey of 1990-1994 and the second on rural northern regions of Pakistan, reported the prevalence of hypertension of 19.1% and 14% in Pakistan [3-4].

Compared with hypertension alone, the risk of developing CVD is two to three times higher in those who have hypertension with diabetes and hyperlipidemia. Treatment adherence and lifestyle modifications to diet, daily activity, and smoking cessation are known to be important in hypertension care [2]. Effective management of hypertension has been a challenge in developing countries. Around two-thirds of the adult population do not get their blood pressure (BP) checked, and, of those diagnosed, only 34% receive appropriate treatment. Furthermore, only 3% of patients with hypertension achieve the BP control target of less than 140/90 mmHg [4].

Most of hypertensive patients in the United States and elsewhere do not reach these target levels of BP, partly because of the poor adherence to prescribed medication and the lack of long-term antihypertensive therapy, as measured by pharmacy refill rates. Although the efficacy of BP reduction in all the major antihypertensive drug classes is comparable, differences in adverse event profiling and long-term tolerability have been found between agents [5]. Treatment guidelines note that the combination of the angiotensin-converting enzyme inhibitor (ACEI) or angiotensin II receptor blockers (ARBs) plus a diuretic or calcium channel blocker (CCB) provides an effective option to reduce the burden of hypertensive patients [6]. Combinations of ACEI/CCB and ARB/CCB incorporate components of monotherapy act via the complementary mechanism of action and thus achieve greater BP reduction with sustainability than when the mono components were administered separately by themselves [7-8]. Tolerability benefits may also be gained from rational drug combinations, such as edema reduction when an ACEI or an ARB is used [9].

Amlodipine/valsartan combination and amlodipine/valsartan/hydrochlorothiazide (Aml/Val/HCTZ) are agents approved for the treatment of hypertension in Pakistan since 2008 and 2011, respectively. Both have demonstrated their good tolerance and provision in clinical trials for effective BP lowering. The real-life effectiveness of combination therapies in developing countries, including Pakistan is, however, largely divided in the literature [10-11]. Aml/Val and Aml/Val/ HCTZ have demonstrated signiﬁcant and effective BP-lowering effect and were well tolerated in several clinical studies conducted in patients with stage 1 and/or 2 hypertension [12]. Real-life observational studies with Aml/Val combination have shown that BP was reduced safely and effectively across all levels of hypertension as well as in patients with isolated systolic hypertension (ISH), mostly with BP targets.

Multiple studies have been conducted outside the country but there is little data locally to assess the effects of this combination for treating hypertension. Another reason is to generate real-world evidence about the efficacy and safety profile of the generic brand Avsar® (PharmEvo Pvt Ltd, Karachi, Pakistan). In the given context, this study was therefore conducted: i) to determine the efficacy of Aml/Val 80/5 mg once-daily dose in reducing mean sitting systolic blood pressure (MSSBP) after eight weeks of therapy; ii) to determine the efficacy of Aml/Val 80/5 mg once-daily dose is reducing mean sitting diastolic blood pressure (MSDBP) after eight weeks of therapy; and iii) to measure the percentage of a participant with a BP change of <139/89 mmHg at the end of eight weeks.

## Materials and methods

This study is an open-labeled observational study carried out in real clinic settings of different tertiary care hospitals and private clinics for a period of 12 months from December 2017 to December 2018 after ethical approval. A total of 900 patients were enrolled in the study out of which 769 hypertensive patients came for follow-up of both genders between the ages of 18 and 70 years whose BP was >139/89 mmHg (not controlled) and was uncontrolled with mono-therapy for minimum last 30 days were included in the study. Pregnant or lactating mothers, patients with secondary hypertension due to any cause, peripheral arterial disease, adrenal disease, or chronic kidney disease patients were excluded from the study. The study was approved by the institutional review board (No: IRB-001/MHS/17). The trial was also registered at www.clinicaltrials.gov. (Identifier: NCT03371797).

After taking written informed consent, all patients were clinically examined and brief history was obtained. BP levels and nonpharmacological parameters on day zero were noted and on the same day, therapy was started. Therapy of all the patients was initiated with Aml/Val (Avsar) for effective control of their hypertension. Patients returned for follow-up after the first week, where their BP, adverse effects, and nonpharmacological parameters were noted. Patients were further followed-up at fourth week and eighth week, where their BP, adverse effects, and nonpharmacological parameters were noted. The primary endpoint for assessment of efficacy was assessed at the end of the eighth week. If BP remained uncontrolled on an initial dose of Avsar, i.e. 80 mg of valsartan and 5 mg of amlodipine, study physician adjusted the dose. Omron M2 basic automated BP monitor that is European approved was used for recording the BP. At each follow-up, three readings of BP were recorded, in a sitting position after five minutes of rest. The measurements were recorded at one-two minute intervals and the mean of three readings was noted. Readings were taken in the whole number, not in decimal (e.g. 180 mmHg not 180.2 mmHg). Free drug was provided for one month as an incentive to patients of being part of this observational study. Patients were allowed to withdraw from the study at any time after informing the study physician. For patients who withdrew prematurely from the study, available data till their last contact were collected by the investigator as defined in the study protocol.

Data were analyzed using SPSS version 23. Demographics and baselines variables were analyzed using descriptive statistics. Repeated measures ANOVA were used to assess the mean changes in BP and pulse at first, fourth, and eighth weeks while chi-square test was used to analyze the safety and efficacy of the given medicines. The significance level was set at 0.05.

## Results

In the present study, there were 769 subjects, 59.0% of them were females, their mean age was 52.01±11.2 years, mean duration of previous treatments was 39.23 ± 45.4 months. The most common anti-hypertensive drug used was CCB while a majority of the patients took 80/5 mg tablets as an initial dose (Table 1).

**Table 1:** Baseline demographics and clinical characteristics of patients (n=769). SD, standard deviation; ACE, angiotensin converting enzyme; ARB, angiotensin receptor blocker; Valsartan, Val; Amlodipine, Aml

Variables		n	%
Gender	Male	315	41.0
	Female	454	59.0
Age	Mean, SD	52.01	± 11.2
Duration of previous treatment, months	Mean, SD	39.23	± 45.4
	ACE inhibitors	85	11.1
	ARBs	110	14.3
	Calcium channel blockers	162	21.1
	Beta blockers	99	12.9
	Alpha blockers	1	0.1
	Diuretics	183	23.9
	Multiple drugs	127	16.6
Co-morbidities	Patient with co-morbidities (n=353)		
	Obesity	101	28.6
	Diabetes mellitus	71	20.1
	Chronic kidney disease	9	2.5
	Chronic heart disease	8	2.3
	Angina	9	2.5
	Previous myocardial infarction	2	0.6
	Recurrent stroke	2	0.6
	Peripheral vascular disease	1	0.3
	Multiple co-morbidities	150	42.5
Initial dose of patients	80/5 mg tablet (80 mg of Val and 5 mg of Aml)	569	73.9
	160/5 mg tablet (160 mg of Val and 5 mg of Aml)	129	16.7
	160/10 mg tablet (160 mg of Val and 10 mg of Aml)	71	9.23

**Figure 1:**
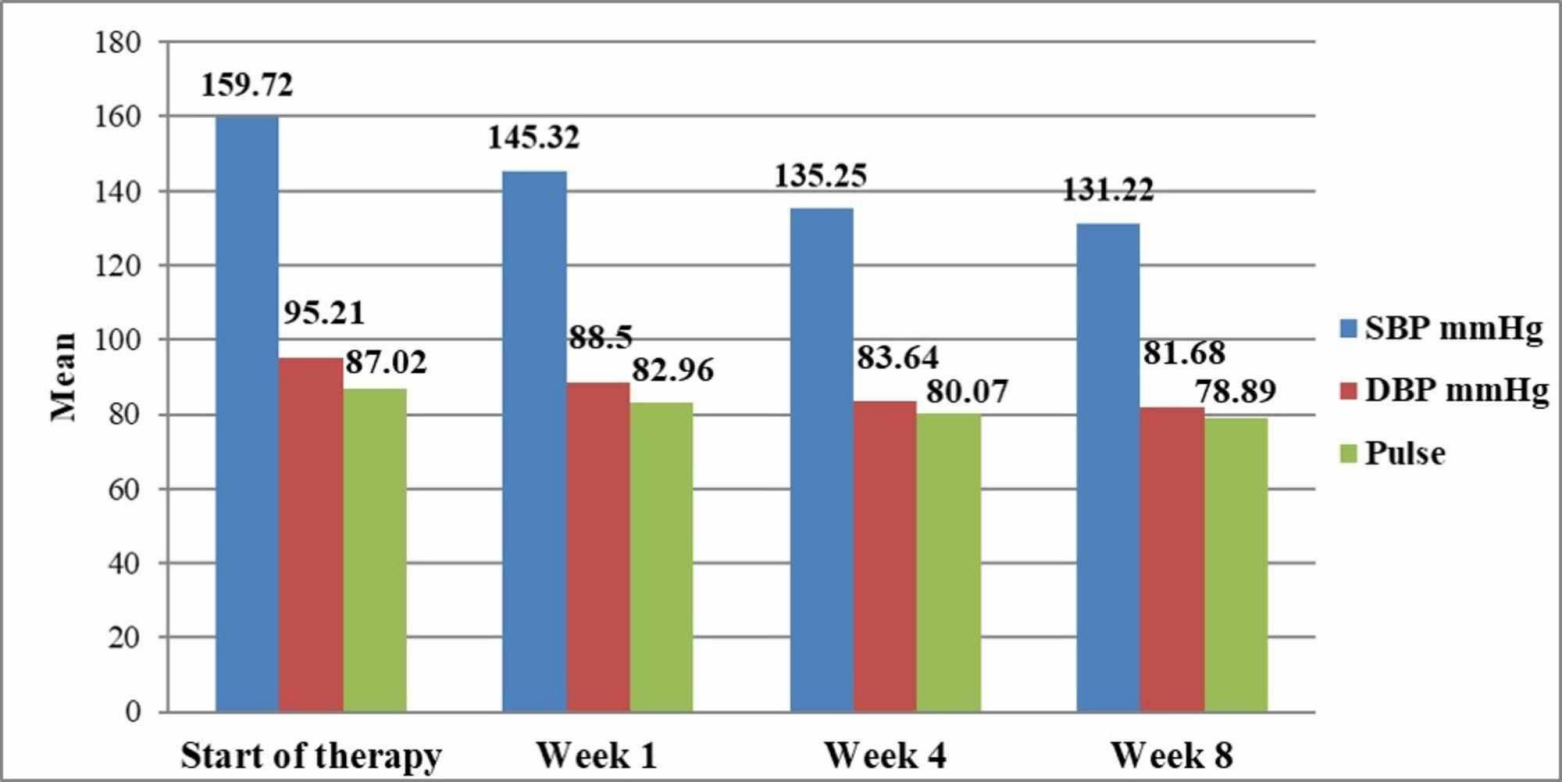
Effect of treatment on SBP and DBP. SBP, systolic blood pressure; DBP, diastolic blood pressure

The study results further revealed that the reduction in MSSBP from start of the treatment to the end of week eight was found to be statistically significant (p<0.01). Moreover, the reduction in MSDBP from the start of the treatment to the end of week eight was also found to be statistically significant (p<0.01). Also, the decrease in mean pulse was found to be statistically significant (p<0.01) (Table 2, Figure 1).

**Table 2:** Effect of treatment on SBP and DBP. *p-value less than 0.05 was considered significant using repeated measure ANOVA SBP, systolic blood pressure; DBP, diastolic blood pressure

Measures	Start of therapy Mean±SD	Week 1 Mean±SD	Week 4 Mean±SD	Week 8 Mean±SD	p-value
SBP mmHg	159.72 ± 18.56	145.32 ± 45.48	135.25 ± 11.78	131.22 ± 39.98	<0.01*
DBP mmHg	95.21 ± 10.39	88.5 ± 28.46	83.64 ± 7.25	81.68 ± 5.85	<0.01*
Pulse	87.02 ± 10.66	82.96 ± 9.61	80.07 ± 8.6	78.89 ± 8.09	<0.01*

**Figure 2:**
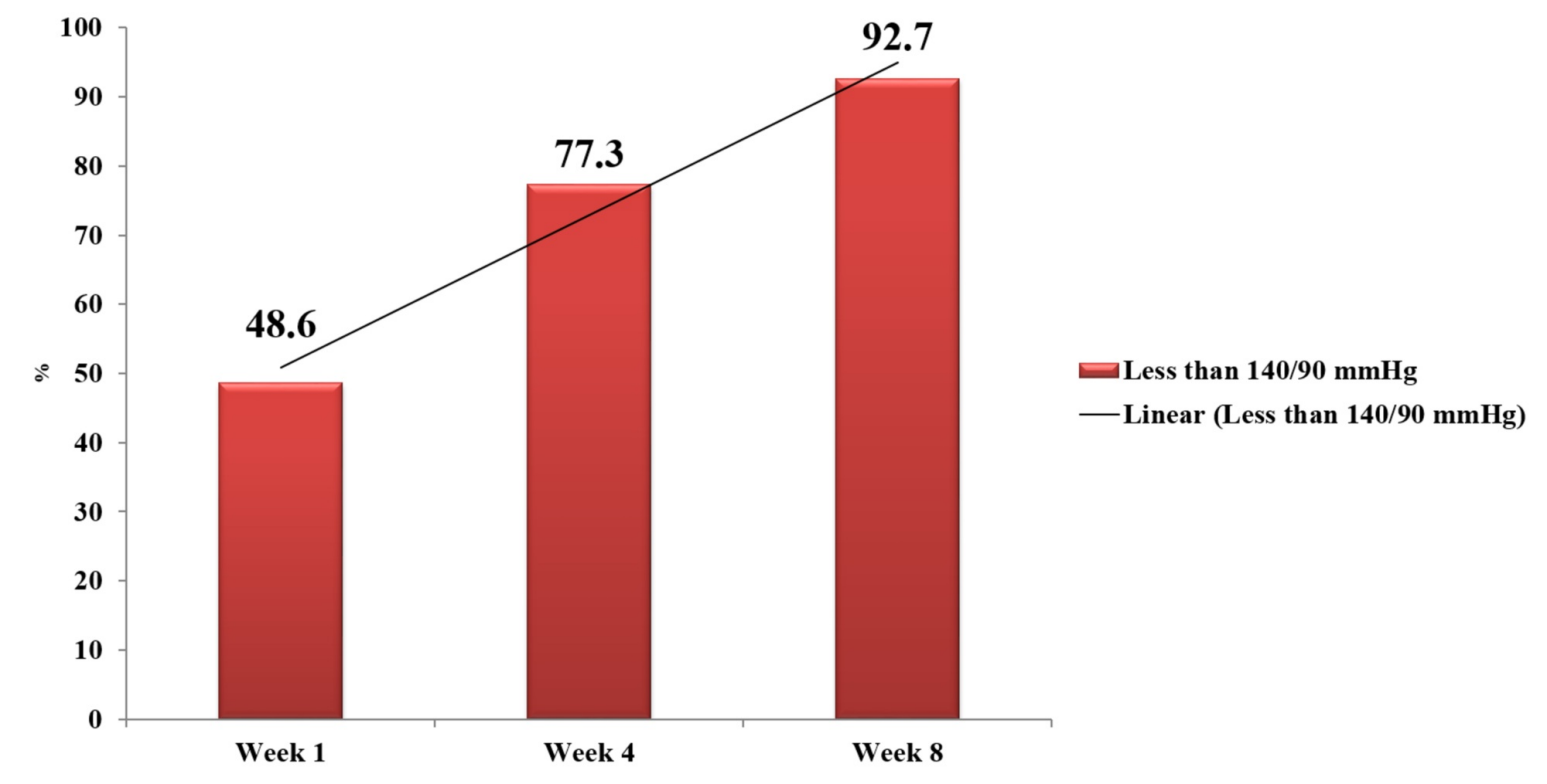
Target BP achievement from Week 1 to Week 8. BP, blood pressure

It was further seen that both exercise and diet significantly reduced the BP level of the participants over the course of the study and with an intervention of eight weeks (p<0.01). The study results further showed that after completion of the week one, 88.6% of the participants achieved the targeted BP with Aml/Val 80/5 mg tablet, 9.1% achieved targeted BP with Aml/Val 160/5 mg tablet, and 2.3% achieved the targeted BP with Aml/Val 160/10 mg tablet. At the end of the fourth week, 82.1% of the participants achieved the targeted BP with Aml/Val 80/5 mg tablet, 14.6% achieved the targeted BP with Aml/Val 160/5 mg tablet, and 3.3% achieved the targeted BP with Aml/Val 160/10 mg tablet. Whereas at the end of the eighth week, 75.6% of the participants achieved the targeted BP with Aml/Val 80/5 mg tablet, 18.5% achieved the targeted BP with Aml/Val 160/5 mg tablet, and 5.9% achieved the targeted BP with Aml/Val 160/10 mg tablet. It has been evident that at all doses of Aml/Val (Avsar), there is a significant BP reduction seen over the course of eight weeks treatment (p<0.01) (Table 3, Figure 2).

**Table 3:** Change in dose with respect to target BP achievement. *p<0.05 was considered significant using Pearson Chi Square test BP, blood pressure; Valsartan, Val; Amlodipine, Aml

Change in dosage	Target BP achieved (less than 140/90)						p-value
	Week 1		Week 4		Week 8		
	n	%	n	%	n	%	
80/5 mg tablet (80 mg of Val and 5 mg of Aml)	155	88.6	202	82.1	192	75.6	<0.01*
160/5 mg tablet (160 mg of Val and 5 mg of Aml)	16	9.1	36	14.6	47	18.5	<0.01*
160/10 mg tablet (160 mg of Val and 10 mg of Aml)	4	2.3	8	3.3	15	5.9	<0.01*

Furthermore, all of the gender, age, presence of any comorbidity, and compliance with the therapy were significantly associated with the achievement of the targeted BP after eight weeks of therapy (p<0.01 for all).

## Discussion

The study results showed that 21.1% of patients were on CCBs, 14.3% were on ARBs, and 11.1% were on ACEs as their primary antihypertensive treatment. Some 16.6% of patients consumed more than one medication. It was further seen that the study medication significantly reduced all of the SBP, DBP, and pulse of the patients at the eighth week of follow up as compared to their baseline values.

A retrospective study by Elliott et al. concluded that among antihypertensive agents studied, valsartan showed the greatest rates of utilization among the subjects [13]. Our study showed that Aml/Val combination helped in reducing BP to the desired levels in majority (92.7%) of the patients.

An observational study by Khan et al. reported that BP goals were achieved by 57% of patients in the Aml/Val group. Most patients rated the effectiveness, compliance, and tolerability as ‘good’ or ‘very good’. The study concluded that Aml/Val was effective and well tolerated for BP reduction in patients of hypertension from Pakistan [14]. The above finding is consistent with our study about the compliance, tolerability, as well as effectiveness, were after eight weeks of treatment; 92.7% of patients in our study achieved the desired target levels of BP.

Another study by Allemann et al. reported that 72.7% of patients on Aml/Val 5/160 mg achieved the desired BP levels by 16th week and 74.8% patients on Aml/Val 10/160 mg achieved the desired BP levels by 16th week. The above results showed the BP-lowering benefits of complementary antihypertensive therapy with Aml/Val in patients with hypertension uncontrolled by previous monotherapy [15]. In contrast to this, our study reported that at eighth week, 75.6% patients achieved targeted BP with Aml/Val 80/5 mg tablet, 18.5% achieved targeted BP with Aml/Val 160/5 mg tablet, and 5.9% achieved the targeted BP with Aml/Val 160/10 mg tablet. Dietary habits, exercise, genetic variability all tend to influence a medication’s desired effect to take place.

A clinical research by Assaad-Khalil et al. demonstrated that the therapeutic goal, SBP response, and DBP response were reported to be 49.3%, 91.1%, and 91.4%, respectively. Aml/Val provided clinically signiﬁcant BP reductions and was generally well tolerated in patients with hypertension [16]. The above findings are in line with our study that also showed greater compliance and effectiveness with Aml/Val.

Chazova et al. in a study reported a significant reduction in BP that was achieved with Aml/Val treatment resulting in a final BP of 129.9/79.3 mmHg. Treatment response increased with increasing initial severity of hypertension and an optimal BP reduction was achieved for all hypertension grades as well as isolated systolic hypertension providing evidence that most hypertensive patients may benefit from Aml/Val combination treatment [17], a finding which is in line with our study.

Patients with uncontrolled hypertension typically require two or more agents to achieve the desired BP levels. Fixed-dose combination therapies with lower doses generally are well tolerated and more effective than higher-dose monotherapy. In a study by Smith et al, patients were randomized to amlodipine, valsartan, combination therapy across the same dose ranges, or placebo. Aml/Val combination therapy was associated with greater BP-lowering effects in the subgroups compared with each respective monotherapy and placebo. These findings were consistent with the primary efficacy analysis results from the overall study populations that combination regimens are generally well tolerated by all patient subgroups [18].

A study by Tung et al. compared two strategies of hypertension treatment in an outpatient, emergency, and inpatient departments: a ﬁxed-dose combination (FDC) of Aml/Val vs. free combinations of ARBs and calcium channel blockers (CCBs) (ARB+CCB group). After a mean follow-up of 15.2 months, the FDC group had signiﬁcantly higher proportion of days covered (80.35% vs. 72.57%), and better persistence (266 vs. 225 days) compared with the ARB+CCB group. The FDC group also had a better major adverse cardiovascular event (MACE)-free survival and decreased rates of heart failure, malignant dysrhythmia, and percutaneous coronary intervention. Compared with free combinations of ARB+CCB, an FDC of Aml/Val improved MACE-free survival and medication compliance and decreased total healthcare costs and hospitalization rates in hypertensive patients [19].

Though our study did not evaluate the side effects of the study medication directly, from an almost complete rate of compliance it may be implied that the serious side effects of the medication were rare during the period of the study.

Our study did not evaluate hypertension in hospitalized patients as well as the cost of the treatment. The other limitation of this study is that the patients enrolled were only followed for a period of eight weeks. Furthermore, it might not be immune to reporting bias. However further studies to evaluate the major adverse events and cost of medication as well as hospitalizations if any are needed to enhance evidence-based use of treatment modalities in hypertension management.

## Conclusions

Our study concluded that Aml/Val (Avsar) combination therapy was very effective in controlling BP upto the levels as recommended by AHA/ACC and ESC/ESH among patients who were uncontrolled with other monotherapy.
